# A comprehensive meta-QTL analysis for yield-related traits of durum wheat (*Triticum turgidum* L. var. *durum*) grown under different water regimes

**DOI:** 10.3389/fpls.2022.984269

**Published:** 2022-09-06

**Authors:** Osvin Arriagada, Agata Gadaleta, Ilaria Marcotuli, Marco Maccaferri, Matteo Campana, Samantha Reveco, Christian Alfaro, Iván Matus, Andrés R. Schwember

**Affiliations:** ^1^Departamento de Ciencias Vegetales, Facultad de Agronomía e Ingeniería Forestal, Pontificia Universidad Católica de Chile, Santiago, Chile; ^2^Department of Agricultural and Environmental Science, University of Bari Aldo Moro, Bari, Italy; ^3^Department of Agricultural and Food Sciences, University of Bologna, Bologna, Italy; ^4^Centro Regional Rayentue, Instituto de Investigaciones Agropecuarias (INIA), Rengo, Chile; ^5^Centro Regional Quilamapu, Instituto de Investigaciones Agropecuarias (INIA), Chillán, Chile

**Keywords:** meta-QTL analysis, yield component, QTL, rainfed, drought, durum wheat

## Abstract

Abiotic stress strongly affects yield-related traits in durum wheat, in particular drought is one of the main environmental factors that have effect on grain yield and plant architecture. In order to obtain new genotypes well adapted to stress conditions, the highest number of desirable traits needs to be combined in the same genotype. In this context, hundreds of quantitative trait loci (QTL) have been identified for yield-related traits in different genetic backgrounds and environments. Meta-QTL (MQTL) analysis is a useful approach to combine data sets and for creating consensus positions for the QTL detected in independent studies for the reliability of their location and effects. MQTL analysis is a useful method to dissect the genetic architecture of complex traits, which provide an extensive allelic coverage, a higher mapping resolution and allow the identification of putative molecular markers useful for marker-assisted selection (MAS). In the present study, a complete and comprehensive MQTL analysis was carried out to identify genomic regions associated with grain-yield related traits in durum wheat under different water regimes. A total of 724 QTL on all 14 chromosomes (genomes A and B) were collected for the 19 yield-related traits selected, of which 468 were reported under rainfed conditions, and 256 under irrigated conditions. Out of the 590 QTL projected on the consensus map, 421 were grouped into 76 MQTL associated with yield components under both irrigated and rainfed conditions, 12 genomic regions containing stable MQTL on all chromosomes except 1A, 4A, 5A, and 6B. Candidate genes associated to MQTL were identified and an *in-silico* expression analysis was carried out for 15 genes selected among those that were differentially expressed under drought. These results can be used to increase durum wheat grain yields under different water regimes and to obtain new genotypes adapted to climate change.

## Introduction

Durum wheat (*Triticum durum* Desf.; 2n = 4 × = 28, AABB) is the 10th most important crop in the world with a cultivated area of 16 million ha and a production of 40 million ton in 2017 (Dahl, 2017). In addition, durum wheat is the most important cereal in the Mediterranean regions since it is deeply connected with the history and culinary tradition in those areas (Martínez-Moreno et al., 2020). This cereal plays a key role in human diet because it is primarily used for making pasta and other semolina-based products, such as frike, couscous, bourghul, and unleavened breads, which are widely consumed in many countries of the world (Sharma et al., 2019). The main producers of durum wheat in the world are Spain, France, Italy, and Greece in southern Europe, Morocco, Algeria, and Tunisia in northern Africa, Turkey, and Syria in southwest Asia, and Canada, United States, and Mexico in North America (Martínez-Moreno et al., 2020), and Argentina and Chile in South America (Colasuonno et al., 2019). Durum wheat is commonly grown in arid and semi-arid regions under rainfed conditions, where the precipitations are scarce and irregular across years (Ayed et al., 2021). The water scarcity combined with high temperatures during grain filling period significantly affects the quality and the yields of durum wheat, causing grain yield losses of up to 50% to farmers (Dettori et al., 2017; Soriano et al., 2021).

According to FAO projections, agricultural production requires to increase about 50% by 2,050 to meet the global rising demand for food (Food and Agriculture Organization of the United Nations [FAO], 2018). In this context, the development of new durum wheat high-yielding cultivars and tolerant to abiotic stresses is highly necessary. Therefore, gaining insight into the genetic basis of the grain yield and their responses to drought stress is an important pre-requisite for improvement of durum wheat genotypes, and plant breeders should look for stable loci to improve yields (Arriagada et al., 2020). The identification of quantitative trait loci (QTL) associated with molecular markers is essential for understanding the genetic basis of important traits, and an effective method for improving selection efficiency in breeding programs (Soriano et al., 2021). Hundreds of QTLs, using both linkage analysis and genome-wide association studies (GWAS), have been mapped into the durum wheat genome, which have been summarized in previous works considering grain quality (Colasuonno et al., 2019; Maccaferri et al., 2019; Marcotuli et al., 2020), and grain yield traits (Maccaferri et al., 2019; Arriagada et al., 2020). Despite these considerable advances in the dissection of the genetic basis for different traits related to quality and yield, only a very small fraction of these QTLs and the associated markers have been utilized in breeding programs (Cobb et al., 2019), due that most of those QTLs have minor effects and their expression is highly affected by the environment, the genetic background and their interactions (Zheng et al., 2021).

Meta-QTL (MQTL) analysis is a powerful tool to facilitate and improve the accuracy of QTL detection which is an important pre-requisite to prioritize and better define the loci and associated molecular markers valuable for marker-assisted selection (MAS). MQTL analysis combines data sets and creates consensus positions for the QTL detected in independent studies for the reliability of their location and effects across different genetic backgrounds and environments (Goffinet and Gerber, 2000; Veyrieras et al., 2007). This method also allows to identify QTL that have pleotropic effects by determining regions of the genome (MQTL) that contain QTLs for different traits (Said et al., 2013). The identification of MQTL has proven to be an effective tool for use in MAS because the MQTL generally have reduced confidence intervals (CIs) and improved phenotypic variation explained. In addition, the MQTL are useful for the identification of promising candidate genes associated with the target traits (Colasuonno et al., 2021; Saini et al., 2021).

In common wheat (*Triticum aestivum* L.), several studies have performed MQTL analysis for root-related traits (Soriano and Alvaro, 2019), adaptation to drought and heat stress (Acuña-Galindo et al., 2015; Kumar et al., 2020), resistance against Fusarium head blight (Liu et al., 2009), grain size and shape (Gegas et al., 2010). In contrast, only two studies have performed MQTL analysis in durum wheat. Recently, Soriano et al. (2021) performed a complete analysis to identified MQTL for quality traits, and tolerance to abiotic and biotic stresses. Previously, Soriano et al. (2017) identified MQTL for phenology, biomass and some yield traits including works from 2008 to 2015. Considering this previous background for purposes of genetic improvement of grain yield, the most appropriate approach is through the simultaneous selection based on grain-yield related traits (Ramazani and Abdipour, 2019). Grain yield is a complex trait governed by hundreds or thousands of loci. Based on this complexity, the genetic dissection of the grain yield inheritance into grain yield components of lower genetic complexity greatly facilitates the identification of the QTL and therefore the MAS efficiency. The aim of the present study was to perform a complete and comprehensive MQTL analysis for grain-yield related traits in durum wheat using articles published in the last 20 years, in order to identify regions of the genome that are useful for durum wheat breeding programs, in which the objective is to increase grain yields of the crop cultivated under different water regimes.

## Materials and methods

### Quantitative trait loci collection and consensus genetic map

An exhaustive literature review was conducted to find studies reporting QTL for grain-yield related traits in durum wheat grown under different water regimes. The QTL identified in each study were classified as follows: (1) QTL under rainfed conditions which correspond to QTL identified under rainfed, water-limited, and drought conditions, and (2) QTL under irrigated conditions that correspond to QTL identified under well-water, optimal, and irrigated conditions as described by the authors in each study. The country, location of each trial, rainfall and type of classification of the QTL is summarized in Supplementary Table 1. A total of 19 traits associated with the two main yield components (grain weight and grains number per unit area) were selected (Table 1). For the MQTL analysis, only studies that showed the following information were considered: (1) type and size of the mapping population, (2) position of QTL (peak position and/or confidence intervals), (3) LOD (logarithm of the odds) score for each QTL, (4) percentage of phenotypic variance explained for each QTL (PVE or *r*^2^). QTL that did not meet these criteria were discarded. Each QTL was treated as an independent QTL, even if some were detected in multiple environments or genetic backgrounds. If the confidence interval (CI, 95%) for the QTL was not reported, it was calculated using the following formulas (Guo et al., 2006):

**TABLE 1:** Traits related to yield components reported in the QTL studies.

Category	Trait	Abbreviation
Grains number per area	Grain number per spike	GNPS
	Grain number per plant	GNP
	Spike number per plant	SNP
	Spikelet number per spike	SLNS
	Grain number per spikelet	GNSL
	Grain yield	GY
	Spike number per m^2^	SNM
	Harvest index	HI
	Grain number per m^2^	GNM
	Grain yield per spike	GYPS
Grain weight	Spike length	SL
	Spike width	SW
	Grain length	GL
	Grain width	GW
	Thousand grain weight	TGW
	Test weight	TW
	Grain perimeter	GP
	Grain area	GA
	Grain weight per spike	GWPS

${}_{C\,I=\frac{530}{N\,x\,R^{2}}}$ for back cross (BC) and F_2_ lines

${}_{C\,I=\frac{287}{N\,x\,R^{2}}}$ for double haploid (DH) lines

${}_{C\,I=\frac{163}{N\,x\,R^{2}}}$ for Recombinant inbred lines (RIL)

where *N* is the population size and *R*^2^ is the proportion of phenotypic variance of the QTL.

The durum wheat consensus map developed by Maccaferri et al. (2015) was used for QTL projection. The map consisted of 30,144 markers, spanning 2,631 cM, and a density marker of 11 markers per cM.

### Projection of quantitative trait loci and meta-QTL analysis

To project the QTL positions detected in the different studies, the original QTL data were projected onto the consensus map following the homothetic approach described by Chardon et al. (2004). The MQTL analysis was conducted with the projected QTL on the consensus map using the software BioMercator V4.2 (Arcade et al., 2004). Two different approaches were performed to MQTL analysis according to the number of QTL per chromosome. When the number of QTL per chromosome was 10 or lower, the approach of Goffinet and Gerber (2000) was carried out. Alternatively, the approach of Veyrieras et al. (2007) was performed when the number of QTL per chromosome was higher than 10. In this case, the best MQTL model was chosen according to Akaike Information Criterion (AIC), corrected Akaike Information Criterion (AICc) and modified AIC with factor 3 (AIC3), Bayesian Information Criterion (BIC) and Average Weight of Evidence (AWE) criteria. The best QTL model was selected when values of the model selection criteria were the lowest in at least three of the five models (Soriano and Alvaro, 2019).

AIC = Akaike information criterion; AICc = corrected Akaike’s information criterion; AIC3 = A variant of AIC that uses 3p as the penalty term.

### Identification of candidate genes

QTL involved in grain-yield related traits in durum wheat grown under different water regimes were projected onto the durum wheat consensus map (Maccaferri et al., 2015) for further comparisons. Gene annotations for the most important marker-trait associations (MTAs) was performed using the high-confidence genes reported for the wheat genome sequence (Svevo browser), available at https://wheat.pw.usda.gov/GG3/jbrowse_Durum_Svevo. The marker locations were defined by flanking marker positions and CI of the MQTL. Gene model regulations were obtained through in-silico expression analysis, using the RNAseq data,^1^ filtered for drought and drought combined with heat stress experiments, with the following identification of the up-regulated genes. Primarily, gene models were identified by the “Chinese spring” annotated sequences and subsequently the homologous genes from “Svevo.” Gene models involved in drought stress during plant development and spike drought during early booting were analyzed using the *in-silico* expression data using database (see text footnote 1) within the markers flanking the MQTL.

## Results

### Quantitative trait loci for yield-related traits under different water regimes

**FIGURE 1:**
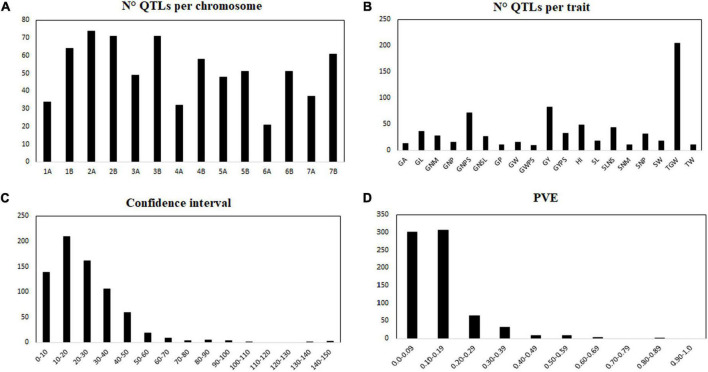
Description of the 724 collected QTLs. Number of QTL per chromosome **(A)**, number of QTL per trait **(B)**, confidence interval **(C)**, phenotypic variance explained (PVE) for each QTL **(D)**. GA, grain area; GL, grain length; GNM, grain number per m^2^; GNP, grain number per plant; GNPS, grain number per spike; GNSL, grain number per spikelet; GP, grain perimeter; GW, grain width; GWPS, grain weight per spike; GY, grain yield; GYPS, grain yield per spike; HI, harvest index; SL, spike length; SLNS, spikelet number per spike; SNM, spike number per m^2^; SNP, spike number per plant; SW, spike width; TGW, thousand grain weight; TW, test weight.

A total of 25 studies identifying QTL for yield components published from 2003 to 2021 based on biparental populations were reviewed in Table 2. The studies comprise 26 different populations with 45 parental lines. A total of 724 QTL distributed throughout all 14 chromosomes (genomes A and B) were collected for the 19 yield-related traits selected. Four hundred sixty-eight QTL were reported under rainfed conditions, and 256 QTL under irrigated conditions (Supplementary Table 2). In general, the number of QTL per chromosome ranged from 21 on chromosome 6A to 76 on chromosomes 2A, with an average of 51 QTL per chromosome (Figure 1A). According to the main yield components, the 53.72% of the QTL (389) were reported for traits related to grains number per area, and the 46.27% of the QTL (335) for grain weight. Specifically, the trait with the highest number of reported QTL was 1,000-grain weight (TGW; 204), whereas the trait with the least reported QTL was grain weight per spike (GWPS; 9), whose both traits are associated with the grain weight (Figure 1B). Confidence intervals (CI) ranged from 0 to 145 cM, with an average of 24.3 cM (Figure 1C). The 19.19% of the QTL had a CI lower than 10 cM, and about half (48.20%) had a CI lower than 20 cM. The proportion of phenotypic variance explained (PVE) for each QTL ranged from 0.007 to 0.83, with an average of 0.138 (Figure 1D). Most of the QTL (608) had a PVE lower than 0.20.

**TABLE 2:** Summary of the QTL studies reviewed including reference, mapping populations, type of population, size, traits, number of QTL collected, water regime, and the number of environments.

References	Cross	Type	Size	Trait	N° QTL	Treatment^a^	Env
Avni et al. (2018)	Zavitan × Svevo	RIL	137	TGW	16	Yes	4
Blanco et al. (2012)	Svevo × Ciccio	RIL	120	TGW, GYPS, GNPS	30	No	5
Desiderio et al. (2019)	Iran_249 × Zardak	RIL	118	TGW, GL, GW, GP, GA	51	No	3
Elouafi and Nachit, 2004	Omrabi 5 × PI600545	RIL	114	TGW, TW	3	No	4
Faris et al. (2014)	Ben × PI 41025	RIL	200	GWPS, TGW, SLNS, GNPS, SL	17	No	2
Fatiukha et al. (2020)	Svevo × Y12-3	RIL	208	TGW	39	Yes	5
Giancaspro et al. (2019)	Saragolla × 02-5B-318	RIL	135	GYPS	29	No	3
Giunta et al. (2018)	Ofanto × Senatore Cappelli	RIL	98	GNSL, SNP, GNPS, SLNS	52	No	2
Golabadi et al. (2011)	Oste-Gata × Massara-1	F_2:3_	151	TGW, GWPS, GNPS, SNM, HI	17	Yes	2
Graziani et al. (2014)	Kofa × Svevo	RIL	249	TGW, GNM, GNPS	64	Yes	16
Maccaferri et al. (2016)	Colosseo × Lloyd; Meridiano × Claudio	RIL	176/181	TGW	5	No	2
Maccaferri et al. (2008)	Kofa × Svevo	RIL	249	GY	10	Yes	16
Mangini et al. (2021)	Liberdur × Anco Marzio	RIL	133	GL, GW, GA, TGW	31	No	3
Marcotuli et al. (2017)	Duilio × Avonlea	RIL	134	GYPS	7	No	2
Milner et al. (2016)	Neodur, Claudio, Colosseo, Rascon/Tarro	MAGIC (RIL)	338	GY	2	No	8
Nagel et al. (2014)	Omrabi5 × Belikh2	RIL	114	TGW, GA, GL, GW	8	No	2
Patil et al. (2013)	PDW 233 × Bhalegaon 4	RIL	140	TW, TGW, GY, SL, SLNS, GNPS, GWPS	44	No	4
Peleg et al. (2009)	Langdon × G18-16	RIL	152	GY, HI	34	Yes	2
Peng et al. (2003)	H52 × Langdon	F_2_	150	GNP, GNSL, GY, SNP, SLNS, GNPS	44	No	1
Rehman Arif et al. (2020)	Omrabi5 × Belikh2	RIL	114	SW, SL, GNPS, TGW, GY, HI	89	Yes	4
Roncallo et al. (2017)	UC1113 × Kofa	RIL	93	HI, GNPS, SLNS, GY, GNP, GNSL, SNM, SNP, TGW	93	No	6
Russo et al. (2014)	Simeto × Molise Colli	RIL	136	GL, TGW, GW	8	No	2
Thanh et al. (2013)	KU7309 × KU8736A	F_2_	144	SLNS, SNP, TGW	5	No	1
Tzarfati et al. (2014)	Langdon × G18-16	RIL	150	TGW	4	No	2
Zaïm et al. (2020)	Four RIL populations*	RIL	576	SNM, TGW, GY	23	Yes	4

*Icamor × Gidara2; Jennah Khetifa × Omrabi5; Algia/Gidara1/Cham1; Omrabi3/Omsnima1//Gidara2; ^a^Irrigated and/or rainfed conditions. Env, environments.

### Quantitative trait loci projection on the consensus map

**FIGURE 2:**
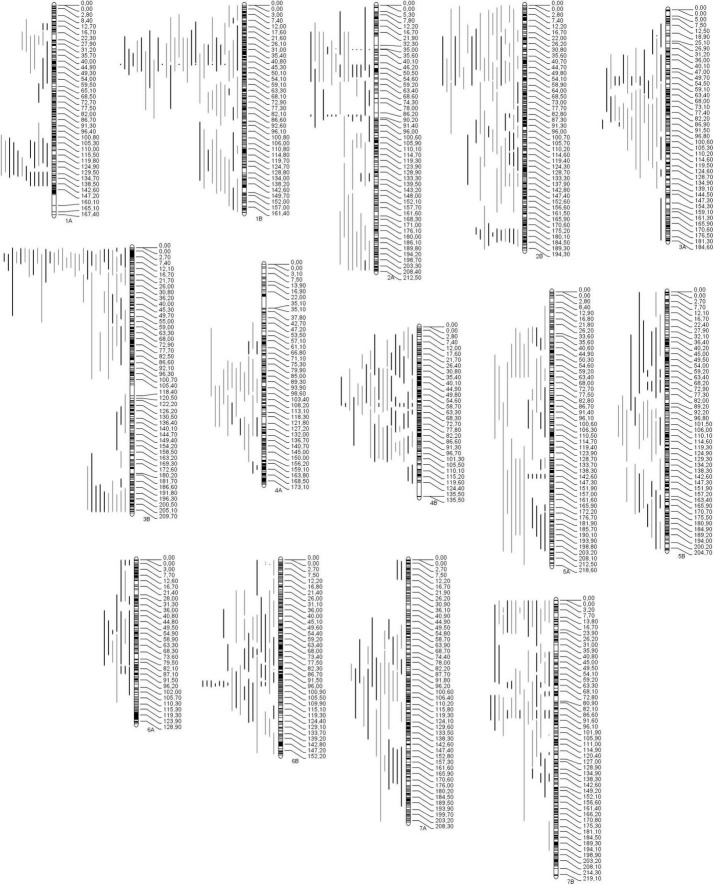
Distribution of the QTL projected throughout all 14 chromosomes (genomes A and B) of durum wheat. QTL were collected for 19 traits related to the main yield components, grains number per area (gray bars) and grain weight (black bars). Black bars within chromosomes represent marker density.

A total of 590 out of the 724 collected QTLs were projected on the consensus genetic map (Figure 2). One hundred ninety-six QTLs were projected under irrigated conditions, and 394 QTL under rainfed conditions (Supplementary Table 3). The remaining QTLs (134) were not projected because they lacked common markers between the original and the consensus maps, and/or the QTL showed low PVE causing a large CI (Soriano and Alvaro, 2019).

**FIGURE 3:**
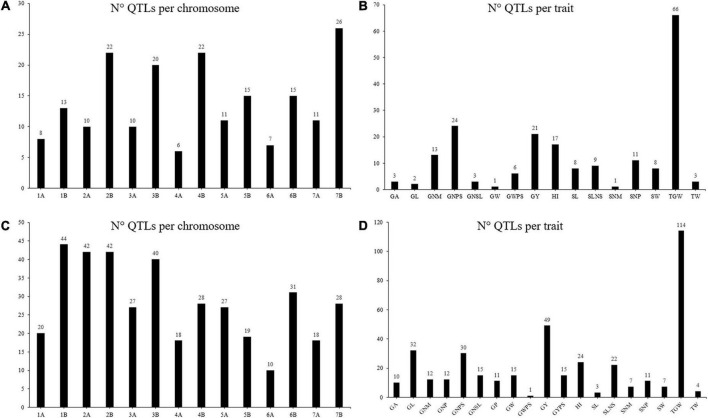
Number of QTL projected under different water regimes. Number of projected QTL per chromosome **(A)** and per trait **(B)** under irrigated conditions. Number of projected QTL per chromosome **(C)** and per trait **(D)** under rainfed conditions.

Under irrigated conditions, the chromosomes 7B (26) and 4A (6) had the highest and lowest number of projected QTL, respectively, with an average of 14 QTL per chromosome (Figure 3A). The trait with the highest number of projected QTL was TGW (66 QTL: Figure 3B). The 49.19% of the projected QTL correspond to the grain weight category while the categories of grains number per area has a total of 99 projected QTL. Under rainfed conditions, the number of QTL per chromosome ranged from 10 on chromosome 6A to 44 on chromosome 1B, with an average of 28 QTL per chromosome (Figure 3C). The trait with the highest number of QTL was TGW (114), while those with the lowest number of QTL were GWPS (1) and SL (3) (Figure 3D). Half of the projected QTL (50%) under rainfed conditions correspond to the grain weight category.

### Meta-QTL detection

**FIGURE 4:**
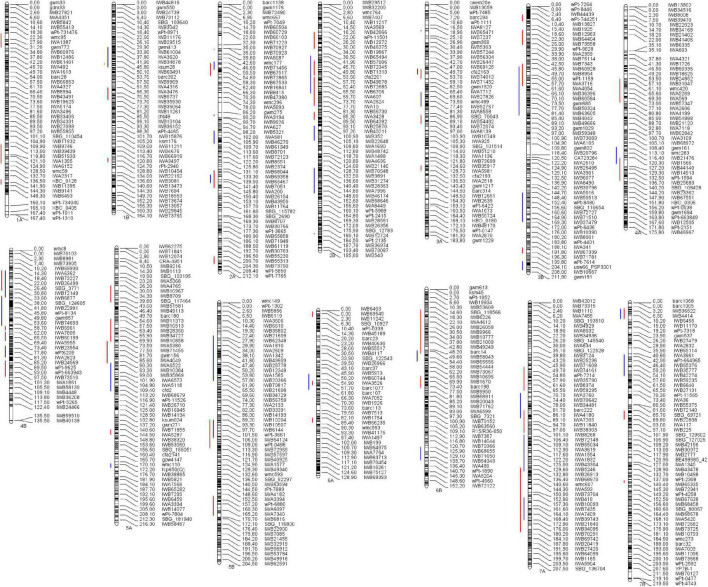
Distribution of the MQTL throughout all 14 chromosomes (genomes A and B) of durum wheat. A total of 76 MQTL were detected under irrigated conditions (blue bars), and under rainfed conditions (red bars). Black bars within chromosomes represent marker density, and to the right is the distance in cM and marker name.

Overall, out of the 590 QTLs projected on the consensus map, 421 were grouped into 76 MQTL. The rest of the QTLs (169) remained as single QTL since they did not overlap with any MQTL interval, the QTL overlapped with more than one MQTL due to their large CI, or because the predicted QTL peaks were not included within any MQTL. Specifically, 28 and 48 MQTL were identified under irrigated and rainfed conditions, respectively (Tables 3,4). Under rainfed conditions, the number of QTL per MQTL varied from 2 on several chromosomes to more than 20 on chromosomes 1B (*YIELD_MQTL1B.1_D*; 47.7 cM) and 3B (*YIELD_MQTL3B.1_D*; 9.8 cM), with an average of 5.89 QTL per MQTL. While under irrigation conditions, the number of QTL per MQTL varied from 2 to 8 on chromosome 3B (*YIELD_MQTL3B.3_I*; 206.94 cM), with an average of 3.75 QTL per MQTL. The number of MQTL per chromosome varied from 1 on chromosome 1B (under irrigated conditions) to 5 on chromosome 2 (A and B) under rainfed conditions. In addition, no MQTL were detected on chromosome 1A under irrigated conditions. The number of traits involved per MQTL ranged from 1 to 9 in the MQTL *YIELD_MQTL1B.1_D* (1B), which also contains the largest number of studies (7). Finally, the CI of the MQTL ranged from 0.12 to 25.96 cM with an average of 6.79 cM, which is significantly lower than the average of CI (24.3 cM) considering the original QTL. Interestingly, in 12 regions of the genome (on all chromosomes except 1A, 4A, 5A, and 6B), the MQTL detected under both water conditions were overlapped (Figure 4 and Table 5).

**TABLE 3:** Characterization of MQTL under irrigated conditions.

Chr	MQTL	Peak (cM)	CI (95%)	N QTL	N studies	Traits	Left marker	Right marker
1B	YIELD_MQTL1B.1_I	42.27	8.43	4	4	HI, SLNS, TGW	IWB31228	IWB57547
2A	YIELD_MQTL2A.1_I	49.12	3.65	3	2	TGW, GNM	IWB54033	IWB73216
	YIELD_MQTL2A.2_I	105.91	5.5	2	2	HI, GW	IWB73852	IWB40575
	YIELD_MQTL2A.3_I	139.11	3.74	2	2	TGW, GY	IWB72154	IWB7051
2B	YIELD_MQTL2B.1_I	56.12	15.49	3	2	TGW, HI	IWB69396	IWB25893
	YIELD_MQTL2B.2_I	102.87	6.33	4	2	GNPS, GWPS, TGW	IWA772	IWB15509
	YIELD_MQTL2B.3_I	140.5	10.56	3	2	HI, GY	wPt-11586	IWB22762
3A	YIELD_MQTL3A.1_I	53.85	2.58	7	2	GNPS, TGW, SW, SL, GY	IWB68183	IWB71974
	YIELD_MQTL3A.2_I	75.82	6.09	2	2	GY, TGW	IWB6187	IWA234
3B	YIELD_MQTL3B.1_I	67.78	19.82	3	3	HI, GNPS, SLNS	wPt-10530	IWB1111
	YIELD_MQTL3B.2_I	160.29	11.99	3	3	HI, TGW, SNM	IWB50437	IWB10030
	YIELD_MQTL3B.3_I	206.94	3.9	8	3	TGW, GNPS, SW, GY, HI	IWB152	IWB8780
4A	YIELD_MQTL4A.1_I	118.62	7.17	3	2	GY, SL, HI	wmc283	IWB1566
4B	YIELD_MQTL4B.1_I	26.92	11.02	4	2	TGW, TW, SNP	wmc710	IWB58189
	YIELD_MQTL4B.2_I	48.41	12.2	2	2	TGW, SL	IWB68116	IWB74693
	YIELD_MQTL4B.3_I	80.85	4.86	3	2	TGW, GY	IWB52747	IWB47175
5A	YIELD_MQTL5A.1_I	102.83	11.68	3	2	TGW, GY	IWB33346	IWB47051
	YIELD_MQTL5A.2_I	173.27	2.11	3	2	TGW, SL	fcp650	IWB68028
5B	YIELD_MQTL5B.1_I	44.52	18.42	4	2	GNPS, SL, TGW	IWB64981	IWB56439
	YIELD_MQTL5B.2_I	100.76	7.57	5	3	GNPS, GWPS, HI	IWB12094	IWB21820
6A	YIELD_MQTL6A.1_I	56.81	6.63	5	3	GNPS, TGW	IWB60744	IWB39171
6B	YIELD_MQTL6B.1_I	53.39	4.71	7	3	SLNS, SNP, HI, TGW	barc14	IWB56048
	YIELD_MQTL6B.2_I	130.89	5.73	3	3	GY, SNP, TGW	IWB7417	IWB19986
7A	YIELD_MQTL7A.1_I	81.89	10.29	4	2	TW, GWPS, GNPS, TGW	IWB27983	IWA4180
	YIELD_MQTL7A.2_I	119.88	14.89	2	2	TGW, GY	IWB1318	IWB29333
	YIELD_MQTL7A.3_I	164.14	9.43	3	3	TGW, SLNS	IWB7435	IWB52522
7B	YIELD_MQTL7B.1_I	9.05	4.46	7	4	HI, GNM, SLNS, GY	IWB30314	IWB6455
	YIELD_MQTL7B.2_I	50.13	9.01	3	2	SLNS, SL, SNP	IWB34143	IWA7589

Chr: chromosome; CI: confidence interval.

**TABLE 4:** Characterization of MQTL under rainfed conditions.

Chr	MQTL	Peak (cM)	CI (95%)	N QTL	N studies	Traits	Left marker	Right marker
1A	YIELD_MQTL1A.1_D	28.78	5.94	5	2	HI, SLNS, GYPS	IWB14137	IWB68107
	YIELD_MQTL1A.2_D	119.16	8.12	8	2	TGW, SNM, GNM	dupw38	barc213
	YIELD_MQTL1A.3_D	141.65	2.48	4	2	TGW	tPt-7724	IWB64946
1B	YIELD_MQTL1B.1_D	47.7	0.53	24	7	GL, TGW, GNPS, GW, GY, GNP, SLNS, GNM, GYPS	IWB8804	IWB12485
	YIELD_MQTL1B.2_D	71.45	5.31	5	3	GNP, GY, GNM, TGW, SNP	IWB51605	IWB6504
	YIELD_MQTL1B.3_D	124.34	2.07	12	4	GY, GNP, TGW, GNSL, GNS, SNP, SLNS	wPt-5034	IWB9116
2A	YIELD_MQTL2A.1_D	35.9	0.38	7	3	GL, TGW, GP, GY, GW	IWB1365	SBG_1442
	YIELD_MQTL2A.2_D	51.89	5.38	4	2	TGW, HI	IWB146	IWB8363
	YIELD_MQTL2A.3_D	88.61	0.84	10	2	SNP, TGW, GW, GL	gwm275	IWA3194
	YIELD_MQTL2A.4_D	139.29	7.4	2	2	TGW, SNP	IWB72154	IWB71648
	YIELD_MQTL2A.5_D	203.93	3.38	8	4	SNP, SLNS, GNP, GA, GNPS, GY	IWB12337	IWB29474
2B	YIELD_MQTL2B.1_D	28.89	7.1	5	3	SNM, GYPS, HI, GNPS	IWB43306	IWB12400
	YIELD_MQTL2B.2_D	51.04	6.27	7	2	GNSL, SLNS, HI, GY	IWB55936	IWB13631
	YIELD_MQTL2B.3_D	69.59	11.86	2	2	SL, GY	IWB46777	IWB53866
	YIELD_MQTL2B.4_D	89.08	3.55	4	2	GP, GNPS, GA, TGW	IWB58691	IWB59170
	YIELD_MQTL2B.5_D	182.93	0.68	5	2	GP, TGW	IWB166	wPt-3755
3A	YIELD_MQTL3A.1_D	21.21	0.8	3	3	TGW, GP, GL	IWB26667	IWB73310
	YIELD_MQTL3A.2_D	55.55	4.2	8	4	TGW, GW, SNM, SW, GNPS, HI	IWB74013	IWB71974
	YIELD_MQTL3A.3_D	91.82	3.84	2	2	GY, TGW	IWB67254	IWB72074
	YIELD_MQTL3A.4_D	130.68	2.52	2	2	TGW, SNP	IWB22148	IWA799
3B	YIELD_MQTL3B.1_D	9.8	3.07	22	5	TGW, GYPS, GNPS, GY, GNSL, GNP, GNM	cfb6045	cfb6021
	YIELD_MQTL3B.2_D	25.42	6.34	4	3	GNP, GY, GYPS	IWB64404	SBG_116252
	YIELD_MQTL3B.3_D	55.17	9.24	6	2	GY, GNPS, TGW, SNM, SW	IWB21831	IWB41640
	YIELD_MQTL3B.4_D	194.66	2.75	3	2	TGW, GNSL	SBG_109559	IWB73613
4A	YIELD_MQTL4A.1_D	66.98	9.9	2	2	GNPS, TGW	IWB2382	IWB18669
	YIELD_MQTL4A.2_D	129.69	9.54	4	3	GY, GL	IWB44140	wPt-1091
4B	YIELD_MQTL4B.1_D	24.39	8.37	4	3	GNPS, GNM, TGW, GW	IWB72973	IWB73302
	YIELD_MQTL4B.2_D	62.12	2.09	12	6	TGW, GNPS, SNM, GY, HI, SLNS, GNSL, GNP	IWB34975	gwm495
	YIELD_MQTL4B.3_D	68.83	4.06	2	2	TGW, GW	IWB17754	IWB62565
	YIELD_MQTL4B.4_D	94.45	6.7	7	3	HI, GNM, GY, TGW	IWB71653	IWB7100
5A	YIELD_MQTL5A.1_D	36.63	7.14	4	3	GNPS, GL, GNM, SW	IWB22285	SBG_117464
	YIELD_MQTL5A.2_D	65.46	9.32	2	2	GY, SLNS	IWB28350	barc40
	YIELD_MQTL5A.3_D	82.78	9.2	2	2	TGW, GNPS	wPt-4248	IWB6959
	YIELD_MQTL5A.4_D	146.45	2.43	5	3	GNP, GW, TGW	IWB55921	IWA4276
5B	YIELD_MQTL5B.1_D	104.63	11.79	6	4	HI, TGW, GY	IWB64691	IWA4094
	YIELD_MQTL5B.2_D	161.97	8.18	3	2	GA, GNPS, GL	IWB162	wPt-3213
6A	YIELD_MQTL6A.1_D	3.1	2.52	2	2	TGW	IWB63240	IWA7288
	YIELD_MQTL6A.2_D	58.04	2	2	2	TGW	IWB73438	IWB66638
	YIELD_MQTL6A.3_D	87.6	2.37	4	3	GY, GW, TW, TGW	IWA8431	barc204
6B	YIELD_MQTL6B.1_D	33.73	17.9	2	2	TGW	IWA5507	gwm508
	YIELD_MQTL6B.2_D	74.09	5.15	5	3	TGW, GA, TW	IWB63659	IWB571
	YIELD_MQTL6B.3_D	101.13	0.12	18	6	SLNS, GA, GNPS, GL, GP, TGW	IWB70152	wPt-3581
7A	YIELD_MQTL7A.1_D	60.93	9.94	3	3	GYPS, TW, SLNS	IWB59818	IWB64911
	YIELD_MQTL7A.2_D	94.92	9.35	7	5	GNPS, GL, SNP, HI, SNM, GY	IWB47576	IWB7751
	YIELD_MQTL7A.3_D	157.17	25.96	3	3	TGW, GY, SL	IWB3767	IWA4620
7B	YIELD_MQTL7B.1_D	9.02	4.55	8	5	TGW, GY, HI	IWB27108	IWB6455
	YIELD_MQTL7B.2_D	89.61	3.87	10	5	GNPS, GYPS, TGW, GY	IWB73443	IWB63652
	YIELD_MQTL7B.3_D	142.8	1.35	4	2	SLNS, TGW, GL	IWB68926	IWB17987

Chr: chromosome; CI: confidence interval.

**TABLE 5:** Regions of the genome where MQTL identified under both water regimes overlap.

Chr	Peak (cM)	Interval (cM)	Left marker	Right marker
1B	47.6	47.1–48.1	IWA107	IWB65324
2A	49.55	46.6–52.5	IWB71456	IWA6478
	138.7	136.0–141.1	IWB72154	IWB64479
2B	50.85	45.3–56.4	wPt-4195	IWB72351
	64.3	58.2–70.4	IWB43195	IWA1664
3A	53.65	52.0–55.3	wmc505	IWB71974
3B	55.95	48.6–63.3	IWA6192	IWB64601
4B	24.2	17.6–30.8	IWB64823	IWB58052
5B	100.55	94.5–106.6	IWA1408	IWB35880
6A	57.95	56.8–59.1	IWB73438	IWB51739
7A	163.65	155.9–171.4	IWB7649	IWB27947
7B	7.55	4.9–10.2	IWB72000	IWB6355

### Candidate genes identification for yield-related traits of durum wheat grown under different water regimes

Candidate genes associated with the MQTL detected were identified using the sequences of flanking markers of the CI launched against the genome browser for both “Svevo” (durum wheat) and “Chinese spring” (bread wheat)^2^ reference genomes. A total of 44 genes were detected and used to determine differentially expressed genes (DEG) up/down regulated under drought/heat conditions using the RNAseq data available at http://www.wheat-expression.com/.

**FIGURE 5:**
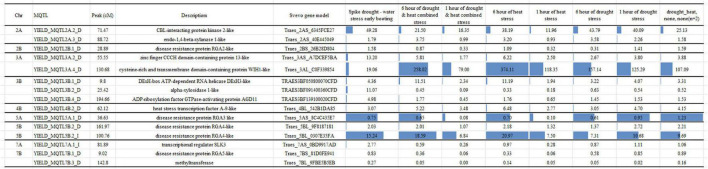
Expressed genes identified in MQTL under irrigated and rainfed conditions.

During the exposure to water stress conditions, the 15 most expressed genes (Figure 5), showing the higher expression level (tmp > 3) were associated to MQTL under both irrigated and rainfed conditions. In particular, the following genes were identified: *CBL-interacting protein kinase 2-like* and *endo-1,4-beta-xylanase 1-like* on chromosome 2A (*YIELD_MQTL2A.2_D* and *YIELD_MQTL2A.3_D*, respectively), *zinc finger CCCH domain-containing protein 13-like* and *cysteine-rich and transmembrane domain-containing protein WIH1-like* all on chromosome 3A (*YIELD_MQTL3A.2_D* and *YIELD_MQTL3A.4_D*, respectively), *DExH-box ATP-dependent RNA helicase DExH3-like*, *alpha-xylosidase 1-like* and *ADP-ribosylation factor GTPase-activating protein AGD11* on chromosome 3B (*YIELD_MQTL3B.1_D*, *YIELD_MQTL3B.2_D*, and *YIELD_MQTL3B.4_D*, respectively), *heat stress transcription factor A-9-like* on chromosome 4B (*YIELD_MQTL4B.2_D*), *disease resistance protein RGA3 like* on chromosome 5A (*YIELD_MQTL5A.1_D*), two *disease resistance protein RGA4-like* on chromosome 5B (*YIELD_MQTL5B.2_D* and *YIELD_MQTL5B.2_I*), transcriptional regulator SLK3 on 7A (*YIELD_MQTL7A.1_I*), *disease resistance protein RGA5-like* and *methyltransferase* on chromosome 7B (*YIELD_MQTL7B.1_D* and *YIELD_MQTL7B.3_D*, respectively).

## Discussion

### Quantitative trait loci for yield component in durum wheat

Increasing productivity under drought stress conditions is one of the main objectives of breeders of staple crops including wheat, due to the need to maintain a sufficient food supply for a growing world population considering the impacts of global warming (Shew et al., 2020). The adaptation to abiotic stress conditions is extremely challenging due to the quantitative genetic basis of the molecular mechanisms adopted by plants to respond to stress (Reynolds et al., 2005). Given that the grain yield components have a quantitative inheritance, and therefore are highly affected by the environment (Nehe et al., 2019), the development of high-yielding varieties must incorporate and accumulate loci associated with yield components that allow them to tolerate the scarcity of water, without affecting significantly their growth and yield. In this sense, numerous studies have been carried out to identify loci associated with the main yield components under irrigated and rainfed conditions in bread wheat (Gupta et al., 2020), and to a lesser extent in durum wheat (Maccaferri et al., 2019; Arriagada et al., 2020).

Grain yield components and their interactions determine the wheat yield (Li et al., 2019). According to our results, among the grain yield components, grain weight is the component most studied in the QTL studies of durum wheat, being TGW the trait most evaluated. This result agrees with the MQTL analyses carried out in bread wheat, in which grains number per spike (GNPS) and TGW are the most evaluated traits under different environmental conditions (Zhang et al., 2010; Gupta et al., 2020). These results can be explained because the main approach to augmenting crop yield is to increase the number and the weight of grains. In fact, TGW is the most important limiting factor affecting wheat yield (Liang et al., 2017). The weight of the grain is the last component of the yield that is formed, and it is highly dependent on the speed and the duration of the grain filling period (Takai et al., 2005), and it is greatly affected by the environment (Li et al., 2019). Therefore, exploring the genetic basis of TGW and its related traits is an effective approach to increase wheat yields (Würschum et al., 2018). According to the distribution of QTLs through the durum wheat genome, the chromosomes with the highest number of QTLs were 2A (76), 2B (71), and 3B (71), whereas chromosome 6A was the one with the lowest number of QTLs (21). These chromosomes consistently contain the greatest number of QTLs for root-related traits (Soriano and Alvaro, 2019), and for quality-related traits, as well as abiotic and biotic stress in durum wheat (Soriano et al., 2021).

### Meta-QTL for yield under different water regimens in durum wheat

In the last decades, many QTL studies have been performed to identify loci associated with grain-yield related traits in bread and durum wheat. However, only a small fraction of these QTLs and the associated markers have been utilized in breeding programs (Cobb et al., 2019), due that most of those QTLs have minor effects and their expression is greatly affected by the environment and the genetic background (Zheng et al., 2021). In this sense, the MQTL analysis has been widely used for collecting data and information of QTL from different populations with different sizes and evaluated under different environmental conditions to identify stable QTL in the plant genomes (Shariatipour et al., 2021). This method allows to identify genome regions (MQTL) implicated in trait variation and reducing the confidence intervals of the QTL. Therefore, the MQTL are useful in marker-assisted breeding programs. In addition, it allows the identification of candidate genes within the MQTL detected in the genome of the target species (Veyrieras et al., 2007).

Several MQTL analyzes have been performed on several important crops such as rice (Islam et al., 2019), maize (Martinez et al., 2016), and barley (Zhang et al., 2017). In wheat, the highest number of MQTL analyzes have been performed in common wheat (Quraishi et al., 2011; Tyagi et al., 2015; Kumar et al., 2020; Zheng et al., 2021). In durum wheat, there are few previous studies of MQTL analysis (Soriano et al., 2017, 2021). In the present paper, we compared the genomic regions involved in durum wheat yield performance under rainfed and irrigated conditions, comparing MQTL in order to identify the most import regions associated to stress tolerance and the candidate genes underlying them. The number of MQTL detected under irrigated conditions is lower than those detected under rainfed conditions, because most durum wheat is sown under rainfed conditions. Twelve regions of the genome overlapping for both rainfed and irrigated conditions. A new MQTL was detected on chromosome 5A (*YIELD_MQTL5A.1_D*), underlying genes activated only during stress conditions. QTL for stress condition were reported also by Soriano et al. (2021) on chromosome 5A. The chromosome 5A seems to have an important role in yield and adaptation trait, and this can be due to the presence of the vernalization genes *Vrn-A1*, favorable alleles for this gene during breeding helps develop spring habit without cold requirements for flowering, thus this can be used as a strategy for introgressing important target traits from non-adapted pre-breeding materials combining the most favorable vernalization alleles (Soriano et al., 2021).

In the present study, an interesting MQTL on chromosome 2A *YIELD_MQTL2A.2_D* (map position 51.86 cM) linked to TGW and HI was co-localized with a MQTL previously described for different traits in durum wheat by Soriano et al. (2021). These authors, in fact, identified a MQTL on the chromosome 2A at 50.8 cM (*durumMQTL2A.3*) associated with traits related to abiotic stress. Specifically, normalized difference vegetation index (NDVI) and chlorophyll content (SPAD) were identified in that genetic region, which are associated to grain yield under drought stress (Cairns et al., 2012). Considering the map position of the two MQTL could be coincident, this strong MQTL for stress and for yield under rainfed conditions can be useful in durum wheat breeding programs, in which the objective is to increase grain yield under drought conditions.

### Identification of candidate genes underlying the stable meta-QTL

This is the first study that identifies and compares wheat MQTL associated with yield components under irrigated and rainfed conditions. Many different genes have been detected and associated to MQTL for yield-related traits grown under different water regimes, some of them related directly to water stress, some others related to secondary mechanism activated by stresses, and finally genes associated to plant development and differentiation.

A gene model identified on chromosome 2A and associated with a MQTL for harvest index and spike length was the *CBL-interacting protein kinase 2-like* involved in the CIPK serine-threonine protein kinases interaction with the activation of the kinase in a calcium-dependent manner. This gene plays a positive regulatory effect in drought stress response, in fact, Wang et al. (2016) found that the over-expression of the *TaCBL-CIPK2* gene confers drought tolerance in transgenic tobacco plants, by regulating stomatal closure. Another detected important gene on chromosome 2A was endo-1,4-beta-xylanase 1-like, involved in the hydrolyzation of the xylan backbones into shorter and soluble xylo-oligo saccharides. The xylanase is strongly expressed in tolerant barley genotype under drought stress for the mobilization of the nutrients from the aleurone layer and endosperm to the developing seed (Hajibarat et al., 2022). Among the gene models detected, different *disease resistance protein RGA* were identified and specifically *RGA3*, and *RGA4* (two different), and *RGA5* on chromosomes 5A, 5B, and 7B under both irrigated and rainfed condition. The *RGA* genes have been identified primarily in response to biotic stresses such as fungal pathogens (Huang and Gill, 2001; Césari et al., 2014) and subsequently for drought stress, due to the interaction with other proteins, which positively affect the ABA biosynthesis in seed germinations (Skubacz et al., 2016) and flag leaves (Onyemaobi et al., 2021).

On chromosome 3A, we reported the *zing finger CCCH domain protein 13-like* which was found to have a function on plant development and tolerance to abiotic stresses such as salt, drought, flooding, cold temperatures and oxidative stress (Han et al., 2021). In addition, we identified *cysteine-rich and transmembrane domain-containing protein WIH1-like*, which is involved in megasporogenesis and germ cell formation from somatic precursor cells (Lieber et al., 2011). A *DExH-box ATP-dependent RNA helicase DExH3-like* (*DExH-box RHs*) gene, which is involved in biotic and abiotic stresses response as well as plant development, was also identified and associated to MQTL on chromosome 3B for most of the traits for grain weight and grain number per area which have been considered. Recently, the relationship between *DExH-box RHs* and temperature stress tolerance has been reported in *Arabidopsis* (Liu and Imai, 2018). Another gene model detected on chromosome 3B was the *α-xylosidase 1-like*, which contributes to maintain the mechanical integrity of the primary cell wall in the growing and pre-growing tissues. Additionally, in *Arabidospis* mutant for *α-xylosidase* the expression of genes encoding specific abscisic acid and gibberellin enzymes was altered in accordance with the aberrant germination phenotype (Shigeyama et al., 2016). Considering that the abscisic acid is involved in plant adaptation to environmental stresses (Audran et al., 2001), we can assume an indirect correlation between the expression of the *α-xylosidase 1-like* in response to water regimes. One additional gene identified on chromosome 3B was ADP-ribosylation factors GTPase-activating protein AGD11, which has a function in diverse physiological and molecular activities and recently an involvement on conferring tolerance to biotic and abiotic stresses in in rice and foxtail millet (Muthamilarasan et al., 2016).

The *heat stress transcription factors* have been detected for MQTL on chromosome 4B. These factors have been largely studied in plants and play a crucial role in response to high temperature, salinity, and drought because they adversely affect the survival, growth, and reproduction by regulating the expression of stress-responsive genes, such as heat shock proteins (Guo et al., 2016).

One gene was identified on chromosome 7A associated with a MQTL for TW, GWPS, GNPS, TGW, the *transcriptional regulator SLK3*, which encodes a regulator of *AGAMOUS* gene and functions together with a repressor of the *AGAMOUS* gene, the *LEUNIG* gene. One experiment in *Arabidopsis* with loss-of-function mutants of the *AGAMOUS*, showed that the repression of the gene by transcriptional regulator SLK3 induced a replacement of the stamens with the petals, and carpels with a new flower (Franks et al., 2001). On chromosome 7B, a *methyltransferase* involved in DNA methylation at cytosine residues and required for gene expression control and genome stability (Thomas et al., 2014), was detected and it correlates to a MQTL for TGW, GL, SLNS. This gene has been characterized and appeared to be express as a response to stress for the regulation of developmental events such as dormancy (Gianoglio et al., 2017), and against stress-inducing treatment, such as damaged proteins (Krzewska et al., 2021).

## Conclusion

In conclusion, the yield components are complex traits controlled by many QTLs with small effect. In this sense, the MQTL studies provide valuable information for QTL fine mapping and key genes for cloning. We performed the first meta-analysis study that identifies and compares durum wheat MQTL associated with yield components under irrigated and rainfed conditions. In this study, a total of 74 MQTLs were detected, where a total of 35 candidate genes associated with drought stress tolerance and yield were identified. A valuable novel aspect of this work was the identification of 12 genomic regions containing stable MQTLs on all chromosomes, except 1A, 4A, 5A, and 6B. Finally, 15 correlated genes that were differentially expressed under drought were reported, which can be very useful in durum wheat breeding programs to increase the grain yields regardless of the water regime used.

## Data availability statement

The datasets presented in this study can be found in online repositories. The names of the repository/repositories and accession number (s) can be found in the article/Supplementary material.

## Author contributions

OA and AS contributed for the conception and design of the study, organized the database and performed all data analysis, and wrote the first draft of the manuscript. AG and IlM performed the analysis and identification of candidate genes and wrote sections of the manuscript. MM, MC, SR, CA, and IvM made important improvements to the manuscript through their revisions and feedback. All authors revised and edited the manuscript, read, and approved the final manuscript.

## Footnotes

1
http://www.wheat-expression.com

2
https://iwgs.org/
